# *Aspergillus* in Children and Young People with Cystic Fibrosis: A Narrative Review

**DOI:** 10.3390/jof11030210

**Published:** 2025-03-09

**Authors:** Emily Chesshyre, Eva Wooding, Emily Sey, Adilia Warris

**Affiliations:** 1 MRC Centre for Medical Mycology, Department of Biosciences, Faculty of Health and Life Sciences, University of Exeter, Exeter EX4 4QD, UKe.sey@exeter.ac.uk (E.S.);; 2 Department of Paediatrics, Royal Devon University Healthcare NHS Foundation Trust, Exeter EX2 5DW, UK; 3 Department of Paediatric Infectious Diseases, Great Ormond Street Hospital, London WC1N 3JH, UK

**Keywords:** *Aspergillus*, pediatrics, cystic fibrosis, CFTR, allergic bronchopulmonary aspergillosis

## Abstract

Cystic fibrosis is a severe, inherited, life-limiting disorder, and over half of those living with CF are children. Persistent airway infection and inflammation, resulting in progressive lung function decline, is the hallmark of this disorder. *Aspergillus* colonization and infection is a well-known complication in people with CF and can evolve in a range of *Aspergillus* disease phenotypes, including *Aspergillus* bronchitis, fungal sensitization, and allergic bronchopulmonary aspergillosis (ABPA). Management strategies for children with CF are primarily aimed at preventing lung damage and lung function decline caused by bacterial infections. The role of *Aspergillus* infections is less understood, especially during childhood, and therefore evidence-based diagnostic and treatment guidelines are lacking. This narrative review summarizes our current understanding of the impact of *Aspergillus* on the airways of children and young people with CF.

## 1. Introduction

Cystic fibrosis (CF) is the most common inherited life-limiting disorder of Northern Europeans, and half of those affected are children [1]. It is caused by the autosomal recessive inheritance of the cystic fibrosis transmembrane conductance regulator (*CFTR*) gene on chromosome 7, leading to a defective CFTR protein [2]. The CFTR protein is present in a variety of epithelial cells, as well as innate and adaptive immune cells, hence the multi-system nature of cystic fibrosis affecting the lungs, gastrointestinal tract, pancreas, liver, and reproductive tract [3]. Over 2000 mutations of the *CFTR* gene have been identified, with the commonest mutation being deletion of phenylalanine at position 508 (F508del). This causes abnormal CFTR protein folding and premature degradation, preventing it from reaching the cell membrane [4]. In the USA and UK, just under 90% of the CF population have the gene variant F508del [5,6], while 80% of the European CF population also have this variant [7]. Key hallmarks of CF are increased sweat chloride, pancreatic insufficiency, and recurrent respiratory infections leading to lung damage and respiratory insufficiency [3]. Bacterial infections are common in children with CF, with the European CF Society Patient Registry (ECFSPR) reporting that in 2023, 53% of children were infected with *Staphylococcus aureus* and 19% with *Pseudomonas aeruginosa* [7]. Childhood infections cause significant lung damage, hence strict infection control practices and aggressive antimicrobial prophylaxis and treatment regimens are the cornerstones of management [3,8,9]. CFTR modulator therapies that correct the underlying defect have led to dramatic improvements in lung function and quality of life measures in people with CF (pwCF) [10,11,12], with predicted improvements in life expectancy [13,14].

*Aspergillus* is the most common fungal infection [1], with *Aspergillus fumigatus* infection being reported in up to 50% of pwCF [15]. Whilst very common, *Aspergillus* infections are far less studied than bacterial infections in children and young people with CF. *Aspergillus* has often been considered as a bystander in the airways of pwCF with the conidia being trapped in the sticky mucus. *Aspergillus*, of which *A. fumigatus* is the most prevalent species, is ubiquitous in the environment and grows at 37 °C. In pwCF, inhaled *Aspergillus* conidia are not effectively cleared from the smaller airways and are associated with exaggerated inflammation and lower lung function [16,17,18,19]. *Aspergillus* infection in early childhood has been associated with structural lung changes and lower lung function [9,20,21]. The impact of widespread CFTR modulator therapies on *Aspergillus* infection in children with CF is yet to be understood, but it is anticipated that *Aspergillus* will continue to play a role in disease morbidity. The following groups are particularly at risk: those who have already developed structural lung disease including bronchiectasis; those who are not able to take CFTR modulator therapy due to an ineligible genotype (≥10% of the European and USA CF population are estimated to have a genotype ineligible for current CFTR modulator therapy [1,22,23]); those who are eligible for CFTR modulator therapy but are not yet on therapy (ECFSPR 2022 data show that 27% of pwCF eligible for CFTR modulators were not on therapy, likely due to individual choice, drug intolerance, and/or lack of reimbursed access in some countries [1,24]); and those who are less responsive to modulators [25]. In this narrative review, we summarize our current knowledge about the clinical epidemiology and impact of *Aspergillus* infections in children and young people with CF and discuss the challenges and knowledge gaps related to the optimal management of *Aspergillus* infections. In addition, a summary of the antifungal immune responses in CF is provided, as these underpin the clinical disease phenotypes observed.

## 2. Antifungal Immunity in Cystic Fibrosis

The characteristics of the immune response underpin the clinical *Aspergillus* disease phenotypes, and in turn have consequences for the diagnosis and treatment of CF-associated aspergillosis. The defective CFTR chloride channel expressed in airway epithelial and immune cells has a profound effect on the host airway environment and the host immune response to pathogens. The low volume of dehydrated secretions, low pH, and anaerobic environment all contribute to the persistence of *Aspergillus* in the airways [26]. Clearance of the inhaled *Aspergillus* conidia in the smaller airways is impaired due to the abundant mucus and impaired ciliary function. The immune response to *A. fumigatus* is often intense yet ineffective, resulting in chronic inflammation that drives lung damage over time.

### 2.1. Innate Immune Response

The innate immune system acts as the first line of defense against inhaled *A. fumigatus* spores. Key elements of the innate immune response include pathogen recognition, epithelial barriers, and innate immune cells such as macrophages and neutrophils. However, these mechanisms are compromised in CF.

#### 2.1.1. Pathogen Recognition

Immune cells identify pathogens through pattern recognition receptors (PRRs), which detect pathogen-associated molecular patterns (PAMPs) and initiate responses to clear infection. *A. fumigatus* PAMPs are primarily components of the cell wall, such as ß-D-glucan, mannan, melanin, galactomannan, and galactosoaminogalactan (GlcNAc) [27]. Several of the PRRs recognizing *A. fumigatus* are defective or have altered expression in the CF lung.

The high level of neutrophil elastase (NE), a protease found in neutrophil granules, in bronchoalveolar lavage (BAL) fluid (BALF) from pwCF cleaves Dectin-1 and -2 resulting in reduced recognition and phagocytic capacity in vitro [28]. Higher levels of CD23 are found in B cells, CD4+ T cells, and NK cells in pwCF with ABPA compared to non-ABPA CF patients [29,30]. CD23 is mainly involved in immunoglobulin E (IgE) production and is also a low-affinity receptor for IgE. Therefore, the increased expression of CD23 is suggested to be involved in the increased susceptibility of ABPA development in CF patients [31]. High CD206 (mannose receptor) expression on CD11b cells is associated with the development of fibrosis and decreased lung function in the CF lung [32,33]. However, macrophages isolated from the BALF of CF patients show downregulated expression of CD206, associated with an impaired ability to phagocytose pathogens [34]. Additionally, in BALF, SP-A appears to be increased in infants and young children with CF, but in older children and adults, SP-A was found to be decreased compared to healthy controls [35,36,37]. The high levels of proteases present in the CF lung, including cathepsin G, proteinase-3, and NE, are known to degrade SP-A and SP-D, and likely contribute to reduced pathogen recognition and phagocytosis [38,39,40]. A polymorphism in the collagen region of SP-A2 has been associated with the development and severity of ABPA [41].

The Toll-like receptors TLR4 and TLR2 show reduced expression on lung tissue sections and epithelial cells isolated from CF patients compared to healthy controls [42,43]. Polymorphisms in TLR9 and TLR3 have been associated with increased susceptibility to ABPA in asthma [44]. The prevalence of these polymorphisms in pwCF is unclear.

The soluble PRR Ptx3 is expressed by alveolar epithelial cells, as well as in monocytes and macrophages, and is important in the recognition of *A. fumigatus*. In pwCF, serum samples showed high Ptx3 levels, while sputum samples showed reduced Ptx3, compared to healthy controls [45]. Low levels of Ptx3 in CF sputa may contribute to the persistence of *A. fumigatus* in CF.

#### 2.1.2. Epithelial Cells

Lung epithelial cells serve as a critical first line of defense against pathogens such as *A. fumigatus,* and can directly engulf and kill pathogens to initiate immune responses [46]. CF epithelial cells show disrupted intracellular signaling, impaired autophagy, and defective pathogen clearance, enabling chronic inflammation that progressively damages lung tissue [47,48]. CF bronchial epithelial cells show impaired killing of *A. fumigatus*, with one study also showing impaired phagocytosis [49,50]. Ceramide, present in higher amounts in CF epithelial cells [51], has been shown to interfere with epithelial cell killing of *A. fumigatus* [51], and inhibition of ceramide by myriocin restores antifungal activity against *A. fumigatus* [52]. In addition, CF bronchial epithelial cells show earlier deterioration of the tight junction barrier in response to *A. fumigatus* compared to CFTR-corrected cells, and this has been shown to be primarily due to gliotoxin [49,53].

#### 2.1.3. Macrophages

CF macrophages show reduced phagocytosis and killing of fungal spores, which is directly linked to defects in lysosomal acidification. Acidification of the lysosome is crucial for fungal killing, but in CF macrophages, lysosomes are abnormally alkaline, even at baseline [54]. The CFTR modulators (tezacaftor (VX-661) and ivacaftor (VX-770)) have been shown to improve lysosomal acidification in vitro and might boost antifungal killing as observed for *Burkholderia cenocepacia* [55]. Treatment of human CF monocyte-derived macrophages with the CFTR corrector Lumacaftor (VX-809) in vitro, resulted in improved bacterial killing [56], but data on fungal killing are still awaited.

Bacterial infection models have shown CF macrophages to be hyperinflammatory, releasing higher levels of inflammatory cytokines and undergoing increased cell death compared to healthy controls [57]. Similarly, macrophages from CFTR-KO mice, and from CF patients, show increased activation of the NOD-like receptor family pyrin domain containing 3 (NLRP3) inflammasome, which contributes to excessive inflammation and release of pro-inflammatory cytokine IL-1B and IL-18 [58,59].

#### 2.1.4. Neutrophils

Neutrophils are abundant in the sputum of CF patients, associated with increased levels of chemokines and cytokines such as IL-8, lipid-mediator leukotriene B4 (LTB4), TNFα, CXCL10, and IL-17 in the airways [60,61,62,63]. High neutrophil counts and excessive airway inflammation have been identified in the BALF of CF infants as young as 4 weeks old, even in the absence of apparent infection [64]. CF neutrophils can effectively phagocytize and kill *A. fumigatus*, but this response is associated with excessive reactive oxygen species (ROS) production, the release of neutrophil extracellular traps (NETs), and increased proteolytic enzymes that contribute to damaging the surrounding tissue [65,66,67,68]. Treatment of CF polymorphonuclear leucocytes (PMNs) with CFTR modulators (ivacaftor and lumacaftor) reduced ROS following *A. fumigatus* challenge in vitro [69].

The CFTR protein is also expressed in the phagosomes and secretory vesicles of neutrophils and is essential for proper degranulation and antimicrobial defense. For instance, in CF patients, dysfunctional CFTR disrupts ion homeostasis in neutrophil cytosol, impairing degranulation [70]. This defect compromises the ability to clear pathogens such as *A. fumigatus*, leading to chronic infections. Treatment of CF patients with CFTR modulator therapy (ivacaftor) restored ion homeostasis, correcting neutrophil degranulation and improving pathogen clearance [70]. Like macrophages, neutrophils from CFTR-KO mice show increased expression of the NLRP3 inflammasome, resulting in increased neutrophil recruitment and IL-1ß release during *A. fumigatus* infection [58].

### 2.2. Adaptive Immune Response

In CF, the adaptive immune response to *A. fumigatus* is dysregulated, with a skewed T cell response and excessive antibody production that further perpetuates inflammation.

#### T Cell Responses

T cells play a crucial role in the immune response to *A. fumigatus* in pwCF, with distinct T helper cell subsets such as Th1, Th2, Th9, Th17, and T regulatory (Treg) cells contributing to various immune functions. In general, Th1 and Th17 cells play a protective role, supporting fungal clearance, inflammatory responses, and neutrophil recruitment [71]. In contrast, Th2 and Th9 cells usually drive allergic inflammation, with the latter playing a significant role in fibrosis and lung pathology. Tregs generally inhibit Th inflammatory responses to control the balance between pro and anti-inflammatory T cell responses [71].

In CF, T cell dysfunction is often characterized by a Th2-skewed response to *A. fumigatus*, resulting in the clinical phenotype of fungal sensitization or ABPA [72]. This skewed response drives IgE production and the release of IL-4, IL-5, and IL-13, which exacerbate allergic inflammation [73]. Flow cytometry studies on peripheral blood of CF patients with ABPA show a skewing towards Th2 cells with a reduction in IFN-y [74]. A recent murine model of ABPA [75] showed that IL-4 and IL-13 produced from CD4+ Th2 cells induced influx and activation of eosinophils causing inflammation. Eosinophils and basophils then produce further Th2 cytokines, such as IL-25, which further augment the Th2 response [76]. Ex vivo studies of bronchial secretions from patients with ABPA have shown that *A. fumigatus* induces the release of extracellular traps (ETs) from eosinophils [77]. These ETs contribute to the thick eosinophilic mucous, causing increased release of toxic granule proteins and damage-associated molecular patterns (DAMPs), causing airway epithelial cell damage [78]. A Th2-skewed response in CF is also associated with increased Th17 activity during fungal infections, which drives excessive neutrophil recruitment and chronic inflammation [79,80]. This Th2-Th17 axis exacerbates immune dysregulation and contributes to the development of persistent lung damage. Furthermore, T cells reactive to *A. fumigatus* in CF patients demonstrate cross-reactivity to other pathogens such as *Pseudomonas aeruginosa*, potentially amplifying inflammation and complicating disease management [81]. The triple CFTR modulator therapy (elexacaftor/tezacaftor/ivacaftor) in pwCF has been shown to decrease the number of antigen-specific T cells, supporting the idea that a defective CFTR protein contributes to impaired adaptive immunity [82]. The co-stimulatory molecule OX40 ligand, which drives Th2 responses, is upregulated in CF patients with ABPA and correlates with low serum vitamin D levels [83]. Ex vivo studies have shown that vitamin D supplementation reduces OX40 ligand expression and Th2-mediated inflammation, offering a potential immunomodulatory strategy [84].

Studies in CFTR-KO mice have demonstrated increased IL-9 production by innate lymphoid cells, promoting Th9 expansion during *A. fumigatus* infection [85]. Furthermore, IL-9 was shown to increase IL-2 production by mast cells and create a positive feedback loop for further Th9 activation. Together, this contributed to allergic inflammation [85]. IL-9 neutralization in these models significantly reduced lung immunopathology and fibrosis, underscoring its therapeutic potential [85]. In lung biopsies from CF patients, IL-9 gene expression was increased compared to controls, and this was linked to an increase in the human calcium-activated chloride channel (hCLCA1), which regulates the expression of mucins. Therefore, IL-9 may also contribute to mucus overproduction in CF [86].

Calcium flux, a critical regulator of T cell activation, is impaired in CF. This leads to excessive calcium entry in CF T cells, resulting in heightened activation of the calcineurin-dependent transcription factor NFAT, which drives hyper-inflammatory responses [87,88]. Further immune dysfunction in CF is linked to altered metabolic pathways. CFTR deficiency impairs indoleamine 2,3-dioxygenase (IDO) activity in mice and humans, disrupting tryptophan metabolism and skewing the Th17/Treg balance [89]. This imbalance leads to heightened inflammation and exacerbates lung damage during *A. fumigatus* infections [89]. Restoring IDO activity has been shown to resolve this excessive inflammation in preclinical models, highlighting the importance of metabolic regulation in CF immune responses [90].

Tregs are reduced in peripheral blood samples from pwCF [91,92]. However, IL-10, an anti-inflammatory cytokine, is elevated in CF T cells, potentially as a compensatory mechanism to limit inflammation [93,94]. While IL-10 reduces Th1 responses and pro-inflammatory cytokines such as IFN-γ in CF, it may also contribute to immune suppression, impairing effective fungal clearance. The triple CFTR modulator therapy (elexacaftor/tezacaftor/ivacaftor) in pwCF led to an increased percentage of Tregs in the blood compared to pretreatment levels [95].

## 3. Non-Allergic *Aspergillus* Infection

### 3.1. Clinical Epidemiology

*Aspergillus* colonization is where airway samples are intermittently or persistently positive for *Aspergillus*, without obvious respiratory symptoms, as opposed to *Aspergillus* bronchitis where airway samples are positive for *Aspergillus* with symptoms of a pulmonary exacerbation unresponsive to antibiotic therapy. The lack of an international consensus definition of persistent *Aspergillus* colonization/infection makes it difficult to compare the clinical epidemiology and outcomes in children with CF. While the Leeds criteria (see Table 1 legend) is the standard developed for *P. aeruginosa* infection in CF [96] and has been used to define persistent *Aspergillus* colonization in children and adults with CF [97], it requires a minimum of four cultures per patient per year to determine whether there is no infection, intermittent infection, or persistent infection, which is a particular challenge in younger children. Other studies in children with CF have used an alternative definition for persistent *Aspergillus* colonization of ≥2 positive cultures in a 6-month [98] or 12-month period [17,99,100,101,102], which is a more practical approach.

The reported rates of positive *Aspergillus* respiratory cultures in children with CF vary due to testing differences (such as frequency of sampling, respiratory sample type, culture techniques, reporting methods, and host factors such as lung disease severity, co-infections, and medications) [103,104]. Studies in children with CF undergoing routine BAL have shown first detection of *Aspergillus* from a median age of 3.2 years [105], and a 9–13.2% positivity rate for *Aspergillus* [9,106]. In a tertiary cohort of children with CF (*n* = 45), an increased sensitivity of *Aspergillus* detection in BAL samples compared to sputum was shown [107]. A total of 29% of 48 BAL samples (taken during respiratory exacerbations) were positive for *Aspergillus*, with only 14% of 976 sputum samples (collected routinely and during exacerbations) being positive [107].

Studies relying on sputum for diagnosis of *Aspergillus* colonization show a higher median age of first detection due to the difficulties obtaining sputum samples from children under 8 years of age. In the CF-SpIT study (*n* = 121 children, aged 6 months to <18 years old), even though 64% of the children recruited were symptomatic, only 11% were able to spontaneously expectorate, and of children under six years of age, only 2% were able to expectorate spontaneously [108]. In a retrospective cohort study of children and young people with CF who had sputum cultures routinely sent at least four times a year, 32% of children had at least one positive *Aspergillus* sputum culture per year, and 11.6% had chronic *Aspergillus* colonization (defined as ≥2 positive respiratory cultures per year) [99]. In another retrospective cohort study in which children with CF also had at least four routine sputum cultures sent per year, 21% developed chronic *Aspergillus* colonization (presence of *Aspergillus* species in ≥2 out of 4 sputum samples/year) [109]. Other sputum studies of adults and children with CF have shown variable rates of *Aspergillus* colonization, with 23.6% showing intermittent colonization [97] and 15 to 18% showing persistent colonization [19,110]. Cough swabs as an alternative for sputum samples have so far not been shown to be useful in detecting *Aspergillus* [111]. These studies exemplify the challenge of comparing the prevalence of *Aspergillus* colonization between studies due to the different populations, different sampling methods, and different definitions of *Aspergillus* colonization.

In the UK, the prevalence of positive *Aspergillus* respiratory cultures in under 16-year-olds with CF has declined in recent years, from 9.4% in 2019 to 3.1% in 2023 (2.1% in <3-year-olds; 2.1% in 4–7-year-olds, 3.4% in 8-to-11-year-olds, and 4.1% in 12–15-year-olds) [6,112]. This decrease in *Aspergillus* colonization coincided with the widespread introduction of CFTR modulators [6,112]. The G551D longitudinal observational cohort study of patients with CF aged ≥ six years old showed a 53% reduction in the odds of *Aspergillus* sputum or oropharyngeal culture positivity when comparing the 2 years prior to the year after the initiation of ivacaftor [110].

To what extent persistent *Aspergillus* colonization without bronchitis causes clinical symptoms and/or lung function decline, either in the short term or long term, is not fully understood. Cross-sectional [103,113,114] and case-control [99] observational studies have shown that children and adults with CF with severe lung disease were more likely to be infected with *Aspergillus* than those without severe lung disease, which may indicate causality in either direction. Several longitudinal observational studies have shown that persistent *Aspergillus* colonization is associated with a greater decline in lung function (*n* ≤ 770) [17,18,19,98,99], but others have not (*n* ≤ 47) [97,101] (Table 1). A retrospective cohort study of 230 pediatric patients with CF found that chronic colonization with *A. fumigatus* was independently associated with an increased risk of hospitalization due to pulmonary exacerbations [17]. Our longitudinal cohort study of 1675 children and young people with CF did not show an association between *Aspergillus* colonization and increased long-term lung function decline [115].

Risk factors for *Aspergillus* colonization that have been identified include oral antibiotics [103], specifically macrolide antibiotics [100,109], and inhaled antibiotics [97,100,103]. It has been proposed that the anti-inflammatory effect of azithromycin may downregulate defenses that would otherwise promote clearance of *Aspergillus,* such as reduced neutrophil chemotaxis due to reduced IL-8 and leukotriene B4 (LTB4) [109].

*Aspergillus* bronchitis is an airway-invasive infection caused by *A. fumigatus* and results in pulmonary exacerbation (including cough, breathlessness, increased sputum production, and chest pain) that is refractory to antibiotic therapy [102,114,116]. It was first described in six adult patients with CF who presented with respiratory deterioration despite antibiotic treatment, whose sputum cultures grew *A. fumigatus* and all responded to antifungal treatment [117]. Prevalence rates of 30% have been reported in adults with CF, but specific data about prevalence rates in pediatric patients are lacking. A single-center observational study in Italy showed that out of 38 children with CF, 1 (2.6%) had *Aspergillus* colonization, and 1 (2.6%) had *Aspergillus* bronchitis [102]. A retrospective German CF registry study calculated an estimated *Aspergillus* bronchitis prevalence of 1.6% in adults and children [114].

The association between *Aspergillus* colonization and the development of *Aspergillus* bronchitis is not clear. Observational data do not support that they occur in a continuum model but instead suggest that they are discrete entities that are not sequentially progressive [15].

**Table 1:** Observational longitudinal studies on long-term effects of *Aspergillus* colonization in children and young people with cystic fibrosis.

Study Type	Population and Follow-Up	Definition of *Aspergillus* Colonization	Outcomes of *Aspergillus* Colonization
Bronchoalveolar lavage studies			
Begum 2022 [118]: Australasian CF BAL study (1999–2017)	*n* = 119 children with routine BAL at 5 years old Median age 5 years (IQR 5.0–5.1) followed up to 12.5 years (IQR 11.4–13.8) 20 (16.8%) *Aspergillus* positive	BAL positive culture for *Aspergillus* at 5 years old	Multivariable analyses showed no association of *Aspergillus* positive BAL at 5 years old with increased lung function decline to adolescence
Breuer 2020 [20]: Australian respiratory early surveillance team for CF surveillance program (2010–2018)	*n* = 330 children with annual CT scans and BAL Median age 3.0 years (IQR 1.2–4.5) 115 children (34.9%) had ≥1 *Aspergillus* positive BAL (*A. fumigatus* accounted for 75% of *Aspergillus* species identified in BAL)	≥1 BAL positive culture for *Aspergillus*	*Aspergillus* ever cultured from BAL during first 5 years of life associated with worse CT PRAGMA score in same year, following year, and at the end of the study at 5 or 6 years old (adjusted for pancreatic insufficiency, age, gender, *CFTR* genotype, and other infections; and baseline CT score for 1 year progression results) *Aspergillus* positive BAL significantly associated with BAL markers of neutrophil inflammation (*p* < 0.001) *Aspergillus* positive BAL significantly associated respiratory admissions risk in the subsequent year (*p* = 0.008)
Harun 2019 [21] Australian CF data registry (2009–2018)	*n* = 156 Median age 5.05 years (IQR 4.99–5.13 years) 28 (17.9%) BAL positive at 5 years old for *Aspergillus*	BAL positive culture for *Aspergillus* at 5 years old	No association of *Aspergillus* positive BAL at 5 years old with lung function decline from 5 to 14 years old (measured by ppFEV_1_) *Aspergillus* positive BAL at 5 years old associated with air trapping on CT chest contemporaneously but no bronchiectasis
Ramsey 2014 [9]: Australian respiratory early surveillance team for CF (2002–2011)	*n* = 56 routine chest CT followed by bronchoscopy at 3, 12, and 24 months old Age at first infant visit mean 0.98 years (0.09 SD) *n* = 7 with ≥1 routine BAL positive for *Aspergillus*	≥1 BAL positive culture for *Aspergillus*	*Aspergillus* positive BAL was associated with reduced lung function two years later (mean −11.3% FEV_0.75_ (95% CI −18.9 to −3.1, *p* < 0.01)) (adjusted for height, gender, age, test type, and test center)
Sputum studies			
Blomquist 2022 [101]: Swedish Registry-based case-control study (2014–2018)	*n* = 437, ABPA excluded Mean age of *Aspergillus* colonized group: 21.5 years (+/−12.8 years SD) Mean age of non-*Aspergillus* colonized group: 23.9 years (+/−13.4 years SD) 64 (14.6%) *Aspergillus * Colonization	≥2 positive airway cultures in 12-month period	*Aspergillus* colonization was not associated with a more rapid lung function decline or increased use of IV antibiotics compared to the non-colonized group pwCF with *Aspergillus* colonization treated with antifungals had a greater lung function decline compared to not treated *Aspergillus* colonization was associated with increased hospital days, use of inhaled antibiotics, IgE levels, and eosinophil counts compared to the non-colonized group
Al Shakirchi 2021 [98]: Retrospective observational cohort study (2000–2015)	*n* = 132 children and adults Mean age 24.7 years (range 6 to 66 years old) *n* = 77 (58%) colonized with *A. fumigatus*	*A.* *fumigatus* in sputum culture ≥1/year	*A. fumigatus* colonization 2 or 3 years in a row was associated with increased lung function decline after adjustment for age and gender and after exclusion of ABPA patients Eradication of *Aspergillus fumigatus* colonization, spontaneously or with treatment, was associated with improved pp FEV_1_
Hector 2016 [18]: Longitudinal 10-year retrospective study (1992–2012)	*n* = 770 children Mean age 9.4 years (+/−8.56 SD) at inclusion *A. fumigatus* colonization according to Leeds categories: 0: 88.6%, 1: 0%, 2: 4.8%, 3: 6.6%	*A.* *fumigatus* in sputum as per Leeds categories *	An association was shown between *A. * colonization Leeds criteria 1 to 3 versus Leeds criteria 0 and lower lung function (*p* < 0.0001) (adjusted for multiple testing)
Noni 2015 [99]: Observational case-control study (1989–2013)	*n* = 80 children Median 14 years (IQR 12–15.75) 20 children with chronic *A. fumigatus* colonization, age, and sex-matched with 60 controls	Case: chronic ≥ 2 positive sputum cultures/year Control: ≥4 negative sputum cultures/year	Patients with chronic *A. fumigatus* colonization had 8.66% lower FEV_1_ (*p* = 0.02) during a 7-year period compared to those never colonized with *Aspergillus* (after adjustment for baseline FEV_1_)
Fillaux 2012 [19]: Longitudinal study (1995–2007)	*n* = 251 children and adults Median age at end of study 16.3 years (IQR 9.8–23.6) 37 (18%) persistent *Aspergillus* colonization	Persistent *A. fumigatus* colonization ≥ 3 positive sputum cultures in 6-months taken at intervals of ≥1 month	Persistent *A. fumigatus* colonization associated with a larger decline in lung function compared to the control group (adjusted OR 10.7 (95% CI 3.0 to 18.3))
De Vrankrijker 2011 [97]: Longitudinal retrospective study (2002–2007)	*n* = 163 adults and children (ABPA excluded) in longitudinal study Age range 7 to 29 years Grouped according to *Aspergillus* cultures: Group 1: *n* = 115, median age 11 years (IQR 7–18) Group 2: *n* = 29, median age 14 years (IQR 10–19) Group 3: *n* = 19, median age 21 years (IQR 1–29)	Presence of *Aspergillus* in >50% of respiratory cultures that year: Group 1: ≤1 year colonization Group 2: 2–3 years colonization Group 3: ≥4 years colonization	No significant difference in adjusted longitudinal analysis in lung function decline between any of the groups
Amin 2010 [17]: Retrospective cohort study (1999–2006)	*n* = 230 Age range 7 to 18 years Persistent *Aspergillus* group mean age 10.9 years *Aspergillus* negative group mean age 11.1 years 37 (16%) persistent *Aspergillus* colonization at baseline	*Aspergillus * colonization: Persistent: ≥2 positive sputum or BAL/year Transient: 1 positive sputum or BAL/year	Both types of colonization were associated with a greater decline in lung function, 3.61% lower ppFEV_1_ (*p* ≤ 0.0001) in persistent colonization group, and 2.14% lower ppFEV_1_ (*p* < 0.001) transient colonization group compared to non-colonized

CF = cystic fibrosis; BAL = bronchoalveolar lavage; IQR = interquartile range; PRAGMA = Perth–Rotterdam Annotated Grid Morphometric Analysis; ppFEV_1_ = percentage predicted forced expiratory volume in 1 s; FEV0.75 = forced expiratory volume in first ¾ of a second; CT = computed tomography; SD = standard deviation; 95% CI = 95% confidence interval; IV = intravenous; ABPA = allergic bronchopulmonary aspergillosis; pwCF = people with CF. * = Leeds categories [96]: 0: Never had any growth; 1: No growth during previous 12 months having previously been positive; 2: ≤50% of months when samples were taken positive; 3: >50% of months when samples were taken were positive.

### 3.2. Diagnostic Tests

*Aspergillus* colonization is diagnosed by culturing the fungus from a respiratory sample. Sputum, either spontaneous or induced, or BAL samples are appropriate specimens to be assessed for the presence of *Aspergillus* spp. The challenge in children of obtaining sputum samples, and the invasiveness of BAL, is a key reason for the lower detection of *Aspergillus* colonization in children compared to adults with CF [119].

To be able to accurately interpret studies reporting *Aspergillus* colonization prevalence, the method of fungal respiratory culture must be considered as there is considerable variation in prevalence depending on the method used. Vergidis et al. showed high-volume cultures to have a yield for *Aspergillus* spp. of 54.2%, compared to 15.7% when using a standard fungal culture [120]. Another study of 216 sputum cultures taken from 77 adults and children with CF showed that the use of high-volume sputum samples, incubated for 3 weeks, had a significantly improved yield of *Aspergillus* compared to standard fungal culture for 5 days with small-volume samples [121].

BAL samples have been shown to be more sensitive in the detection of *Aspergillus* in CF compared to sputum samples [107], which may in part be due to the high volume available for culture of BAL compared to sputum samples. Bronchoscopic characteristics of *Aspergillus* bronchitis are mucoid impaction and bronchial plugging from viscid sputum, and bronchial erythema and/or ulceration may be a feature [122].

In *Aspergillus* bronchitis, *Aspergillus*-specific immunoglobulin G (sIgG) antibodies are raised in addition to positive respiratory cultures. Serological markers of *Aspergillus* sensitization (AS), total IgE, and *Aspergillus*-specific IgE are not raised (Table 2).

The use of galactomannan (GM) and *Aspergillus* polymerase chain reaction (PCR) for diagnosing CF-associated aspergillosis has hardly been studied [116,120], and no pediatric data are available. A recent study comparing ITS2 (internal transcribed spacer 2) sequencing analysis for identifying fungal pathogens to conventional culture of BAL and induced sputum from 23 children with CF showed that in over 50% of the cohort, fungal pathogens were not detected by routine microbiology culture [123].

The diagnosis of *Aspergillus* bronchitis is strongly supported by features of structural lung damage shown on chest computed tomography (CT) [124], though these are non-specific, with similar features occurring in CF lung disease itself and bacterial infection (Table 2). *Aspergillus* isolation from BAL samples was associated with increased air trapping on high-resolution CT-chest in children with CF [20,21]. In a prospective observational study of 32 pwCF (age range 13 to 32 years; ABPA excluded), those with persistent *Aspergillus* colonization (defined as ≥2 positive sputum cultures at least 4 weeks apart in the year prior to inclusion), compared to those without, had increased severity of bronchiectasis and mucous plugging shown on CT [125]. Chest magnetic resonance imaging (MRI) has the advantage of no radiation exposure to the child and is a useful alternative in centers that have experience in this modality for children with CF (Table 2) [124].

**Table 2:** Key clinical and diagnostic features of *Aspergillus* colonization, *Aspergillus* bronchitis, *Aspergillus* sensitization, and ABPA in cystic fibrosis [30,116,124,126].

	Non-Allergic Infection		Allergic Infection	
	*Aspergillus* Colonization	*Aspergillus* Bronchitis	*Aspergillus* Sensitization	ABPA
Clinical symptoms	None	Productive cough, thick sputum, breathlessness, chest pain refractory to antibiotic therapy	None or mild wheeze	Characteristics: wheeze, cough, exercise intolerance, exercise-induced asthma, decline in pulmonary function, chronic productive cough, increased sputum, breathlessness, pleuritic chest pain, hemoptysis
Diagnostic tests				
*Aspergillus* culture *Aspergillus* + PCR Total IgE *Aspergillus* sIgE *Aspergillus* sIgG Galactomannan Serum eosinophilia	+ + Normal Normal Normal/Raised Normal No	+ + Normal Normal Raised Raised No	− − Normal/raised Raised Normal Normal Normal/raised	+/− +/− Raised: ≥500 IU/mL * Raised: ≥0.35 kUA/L * Raised Raised Raised: ≥500 cells/µL * (could be historical)
Imaging characteristics				
CT scan	Bronchial wall thickening, low attenuation regions, some mucous plugs	Bronchiolitis pattern with centrilobular nodules, tree-in-bud, bronchial wall thickening, bronchiectasis, ground glass opacities, peribronchial consolidation	No change	Central bronchiectasis with mucous plugs and high-density mucus
MRI scan	Low intensity regions, T2 hyperintense plugs	Bronchial wall thickening, T2 weighted imaging hyperintense peribronchial consolidation peripheral mucous plugs	No change	Bronchial wall thickening, T2 hyperintense mucous plugs (in 30% of cases inverted mucus sign, mucus with high T1WI and low T2WI signal)

ABPA = allergic bronchopulmonary aspergillosis; PCR = polymerase chain reaction; IgE = Immunoglobulin E; sIgE = specific Immunoglobulin E; sIgG = specific Immunoglobulin G; IU/mL = international units per milliliter; kUA/L = kilounits per liter; cells/µL = cells per microliter; CT = computed tomography; MRI = magnetic resonance imaging; T1WI = T1 weighted images; T2WI = T2 weighted images. * cut-off as per 2024 updated International Society for Human and Animal Mycology (ISHAM) consensus criteria [126].

## 4. Allergic *Aspergillus* Infection

### 4.1. Clinical Epidemiology

A number of international clinical consensus criteria have been developed to aid in the diagnosis of ABPA [30,126,127]. Despite the differences between these criteria, for example the inclusion or not of eosinophilia, when the CF Foundation and the first ISHAM-ABPA working group criteria were compared in a group of 86 children and adults with CF, no differences were found in the prevalence of ABPA [128]. AS is characterized by raised *Aspergillus*-specific IgE, without the other features of ABPA (Table 2) [30,116,124,126].

A meta-analysis in 2015 reported a pooled prevalence of ABPA in CF of 8.9% in children and 10.1% in adults, and AS in 41.6% in children and 36.1% in adults with CF [129]. More recently, data from the ECFSPR showed a 4.6% prevalence of ABPA in adults with CF and 1.8% in children in 2022 [1]. UK CF Registry (UKCFR) data show a prevalence of ABPA in children < 16 years old of 1.6% in 2023, reduced from 3.5% in 2019 [6,112]. The lower prevalences observed in recent years are likely one of the advantages of treatment with CFTR modulators.

The main reported predisposing factors for ABPA in children with CF are atopy, reduced lung function, chronic respiratory infections, and poor nutritional status [109,130,131,132,133]. A retrospective case-control study of 150 children with CF, matched by age of diagnosis, demonstrated significantly increased duration of prior intravenous anti-pseudomonal antibiotics in those with ABPA (*p* < 0.05) compared to those who did not, though this may be representative of increased disease severity in the ABPA group [134]. In mice models, alterations in the gut mycobiome with antibiotics have been linked to increased CD4 T cell-driven allergic responses to intranasal *Aspergillus* in the lung [135] and enhanced asthma severity in mice [136], but no studies on the gut mycobiome–lung axis have been conducted in CF mouse models [137]. CF patients with the F508del mutation have an increased incidence of ABPA compared to those with milder mutations [133,138], though a recent retrospective study in Greek CF patients did not show any differences between the underlying *CFTR* genotype and ABPA or *Aspergillus* colonization [139]. A meta-analysis of four case-control studies in the non-CF population (*n* = 79 ABPA and 268 controls) showed increased odds of *CFTR* mutation in the ABPA patients compared to controls (OR 10.39; 95% CI, 4.35–24.79) [140]. Genetic polymorphisms in immune genes and HLA loci have been associated with either an increased risk of or protection against ABPA [141,142,143].

In a retrospective case-control study (*n* = 93) of pwCF matched on sex, age (median age 9 and 12 years in AS and AS control groups, respectively), and weight, an association between AS and both length of *P. aeruginosa* colonization and increased doses of inhaled corticosteroids was found, which may represent more advanced CF lung disease in those with AS [144].

The clinical hallmark of ABPA is predominant wheeze. Other features are often indistinguishable from other causes of pulmonary exacerbations and include a chronic productive cough, breathlessness, increased sputum production with a characteristic golden-brown color, pleuritic chest pain, hemoptysis, exercise-induced dyspnea, and reduced lung function [30,145]. There may be systemic features such as a low-grade fever, weight loss, and malaise [126]. 

Observational studies demonstrate that ABPA is associated with poorer lung function [19,113,116,146,147,148], though studies show conflicting results as to the long-term impact [133,149]. An ECFSPR registry study of children with CF aged 6 to 17 years old (*n* = 3350) showed 1.47% lower lung function (ppFEV_1_ (percentage predicted Forced Expiratory Volume in 1 s), *p* = 0.003) at study entry in patients with ABPA, but no increased decline in lung function over the 3-year follow-up period compared to those without ABPA [149]. Our longitudinal cohort study showed that ABPA in children and young people with CF (*n* = 1675) was associated with long-term lung function decline (mean difference in ppFEV_1_ between groups −0.5% (−0.6 to −0.3, *p* < 0.00001)) and body mass index (BMI) decline (mean difference in percentile BMI between groups −0.8% (−1.1 to −0.6, *p* < 0.00001)) over the subsequent 10 years [115].

Patients with AS may be asymptomatic or have a mild wheeze [116]. Although ABPA is more common in patients with AS, there is not necessarily a clinical progression from AS to ABPA. In the cohort study by Baxter et al. [116], 17% of 146 adults with CF and *Aspergillus* switched phenotypes during the 2-year follow-up period. Observational studies have shown an association, though not necessarily causal, of poor lung function in CF patients with AS, compared to CF patients without AS [116,150,151]. ABPA and AS may also be associated with other allergic *Aspergillus* diseases in CF, such as allergic *Aspergillus* sinusitis [152].

### 4.2. Diagnostic Tests

The laboratory diagnosis of allergic *Aspergillus* disease is based on elevated serological responses to *A. fumigatus* (*Aspergillus*-specific IgE) and total IgE (Table 2). Elevated total IgE and *Aspergillus*-specific IgE are markers of both AS and ABPA in children and adults with CF but do not differentiate ABPA from AS [129]. Uniform cut-off levels have not previously been established, mainly because the majority of studies on ABPA have been conducted in patients with asthma rather than CF [127,131,153,154,155]. The revised ISHAM-ABPA working group [126] recommends a cut-off for serum total IgE of ≥500 IU/mL, as this has shown to be more sensitive compared to a cut-off of ≥1000 IU/mL in studies in adults with asthma [155]. *Aspergillus*-specific IgE ≥ 0.35 kUA/L on fluorescent enzyme immunoassay (EIA) has shown to be 99–100% sensitive for ABPA screening in adults with asthma, in comparison to *Aspergillus* skin prick test (SPT) with only an 88–94% sensitivity [155]; therefore, screening with *Aspergillus* SPT is only recommended if *Aspergillus* sIgE is not available [126]. It is important to be aware when using any of these criteria in children and adolescents with CF that they are based on data from adult patients, often those with asthma rather than CF. There is a need to evaluate the validity of these criteria in pediatric CF patients. As the clinical symptoms of ABPA are non-specific, international consensus guidelines for ABPA recommend annual screening with total IgE to enable early detection and treatment [30,126].

Specific IgE responses to *A. fumigatus* recombinant antigens (rAsp) can help differentiate sensitization from ABPA in CF [156], with sIgE to rAsp f3 increasing significantly during ABPA flares in pediatric CF patients [74]. A meta-analysis in patients with asthma and CF showed that a combination of sIgE against rAsp antigens f1, f2, f3, f4, and f6 was helpful in the diagnosis of ABPA [157].

*Aspergillus*-specific IgG is raised in ABPA and not in AS [30,116]. A meta-analysis of studies evaluating *Aspergillus*-specific IgG in ABPA in asthma showed the superiority of immunoassays compared to immunoprecipitation in terms of sensitivity [158]. More recently, lateral flow assays have become available to measure *Aspergillus*-specific IgG in adults with chronic *Aspergillus* disease, including ABPA in the UK [159] and France [160]. Studies are needed to identify the optimal cut-off for *Aspergillus*-specific IgG immunoassay and the value of the lateral flow assay in children with CF.

Peripheral blood eosinophilia (≥500 cells/µL as defined from studies in asthma), though not specific for ABPA, is a criterion in the diagnosis of ABPA in the ISHAM-ABPA working group clinical consensus criteria [126,161].

Radiological abnormalities in ABPA are non-specific and overlap with many of the hallmarks of CF itself including pulmonary infiltrates, bronchiectasis (often central), pulmonary fibrosis, and obstructive lung disease (Table 2) [124]. The revised ISHAM-ABPA working group has recently outlined an updated radiological classification of ABPA [126]. The patient variation in stages of presentation and progression is not understood [153]. Patients may present at any of the stages, and transition from stage 1 through to stage 5 is not necessarily sequential.

Fractional exhaled nitric oxide (FE_NO_) may be a useful test in differentiating ABPA from AS. A prospective observational cohort study of 62 children and young people with CF showed that the mean FE_NO_ and peripheral blood eosinophilia were both significantly raised in ABPA compared to AS [162].

Thymus- and activation-regulated chemokine (TARC) may be a useful biomarker to differentiate ABPA from AS or non-allergic *Aspergillus* disease [163]. In a small study of 48 pediatric CF patients, TARC levels were significantly higher in those with ABPA compared to those without ABPA, with a greater diagnostic accuracy than total IgE or sIgE against recombinant fungal antigens [164]. The basophil activation test (BAT) and lymphocyte stimulation test (LST) are functional immunological tests that could be useful diagnostic tests for ABPA, as shown in a small study including 29 adults with CF [165]. The BAT, which detects immediate hypersensitivity, correlated with impaired lung function, while the LST, which detects delayed hypersensitivity, was higher in patients who went on to develop ABPA 3 months later [165]. A small study on 76 adults and children with CF showed that the BAT test was able to discriminate between ABPA from AS and *Aspergillus* colonization over time and was not affected by antifungal or corticosteroid treatment [166]. Larger studies are required to validate the utility of these new immunological tests in the diagnosis of allergic *Aspergillus* disease in children with CF.

## 5. Management of *Aspergillus* Disease in Children with CF

Optimum management of both allergic and non-allergic *A. fumigatus* disease in children and young people with CF is not well characterized [167,168]. There are no randomized controlled trials (RCTs) in adults or children with CF-associated ABPA [169,170]. Oral azole antifungal treatment was associated with improved lung function in three small noncomparative case series of children with CF and ABPA, comparing before and after voriconazole treatment (*n* ≤ 14) [171,172] and before and after posaconazole treatment [173] (Table 3). A retrospective case-control study of children and adults with CF (65 with ABPA, 127 sex and lung function-matched controls) showed no difference in long-term lung function decline between ABPA patients treated with itraconazole and short courses of steroids versus matched controls without ABPA [174].

The lack of evidence on how to manage *Aspergillus* colonization and infection leads to different clinical practices. A UK survey demonstrated highly variable antifungal management between CF professionals [168], with many clinicians not treating asymptomatic *Aspergillus* colonization, while other clinical guidelines recommend a 2-week course of posaconazole [175]. A recent international survey of CF clinicians in 35 countries across several continents showed a comparable picture, with high variability in the use of antifungals between countries and continents [176].

An RCT comparing itraconazole versus placebo in CF patients with *Aspergillus* colonization was unable to reach any conclusions due to sub-therapeutic itraconazole level [177]. The cASPerCF trial on optimal dosing of posaconazole in children and young people with CF and *Aspergillus* colonization was prematurely terminated due to poor patient recruitment, the impact of the COVID-19 pandemic, and the rollout of CFTR modulator therapy [178].

For the treatment of allergic *Aspergillus* disease, targeting the inflammatory response with systemic corticosteroids has long been the cornerstone of treatment of ABPA [179,180,181,182,183]. However, side effects of systemic corticosteroids, particularly osteopenia, poor growth, and hyperglycemia, restrict the prolonged use of high-dose oral steroids, particularly in children with CF. No RCTs have been conducted to determine the optimum dose and length of corticosteroid therapy for ABPA in adults or children with CF [184]. The ISHAM-ABPA consensus criteria [126] recommend as first-line treatment for acute ABPA low-to-moderate dose oral prednisolone (0.5 mg/kg/day for 2–4 weeks, tapered and completed over four months) as monotherapy, with first-line monotherapy with systemic glucocorticoids also being recommended by other consensus criteria [30]. Meanwhile, a combination of systemic glucocorticoids and antifungals from the outset is recommended by other guidelines [185] and has been shown to be common clinical practice in the UK CF clinician survey [168]. Monthly IV methylprednisolone alone, or with azoles, has been used to limit the toxicity associated with daily corticosteroids with some success in the first episode of ABPA in nine children and adults with CF [186], and in refractory ABPA in CF [187]. However, significant side effects were reported in the latter study.

Azoles are recommended in all the consensus guidelines as combination treatment with glucocorticoids, or as monotherapy if glucocorticoids are contraindicated, for ABPA. The use of azoles in children with CF can be challenging. Poor oral bioavailability of azoles is a key issue in children with CF, which makes therapeutic drug monitoring (TDM) essential [174,177]. The strong inhibition of cytochrome p450 3A4 by azoles alters the metabolism of CFTR modulators (requiring a dose reduction and TDM) and glucocorticoids (inhaled steroid doses may need to be decreased) [185,188,189].

Itraconazole is the first-line recommended azole by the CF Foundation Consensus [30], the IDSA guidelines [185], and the ISHAM-ABPA revised consensus guidelines [126]. Itraconazole has poor oral bioavailability in children with CF [190]. However, a newer formulation of itraconazole with increased bioavailability, SUBA-itraconazole^®^, was evaluated in a retrospective cohort study of 19 children (16 with CF and ABPA), where 59% achieved therapeutic level, 81% demonstrated a decline in total IgE, and it was well tolerated (Table 3) [191].

Voriconazole is an alternative, as it is generally better tolerated and better absorbed than itraconazole. Two retrospective observational studies involving children and adults with CF and ABPA showed that a combination of oral corticosteroids and voriconazole led to a reduction in oral corticosteroid doses required, as well as a reduction in total IgE and improved FEV_1_ [171,172]. A case report describes a 12-year-old boy with ABPA and CF unresponsive to itraconazole and oral corticosteroids, who showed a clinical response to voriconazole with oral corticosteroids with improved lung function and reduced *Aspergillus* sIgE [192].

Posaconazole has fewer drug interactions, is easier to achieve therapeutic levels, is dosed once daily, and has better palatability than the older azoles [173]. A prospective open-label observational study showed that posaconazole led to an improvement in symptoms and lung function in 14 children with ABPA and CF [173]. Another observational study of 32 adults and children with CF and ABPA showed that posaconazole treatment (but not itraconazole or voriconazole) was associated with a significant reduction of *Aspergillus*-specific IgE (Table 3) [193].

Monitoring treatment response is multifactorial, incorporating clinical improvement in symptoms (objectively measured with the Likert score or visual analog scale ≥ 50% improvement), immunological improvement (such as 20% reduction in *Aspergillus* sIgE), and radiological improvement [126]. For patients who are not improving with standard therapy with steroids and oral azoles, the addition of nebulized amphotericin has been used, as shown in a case series of children and adults with CF and ABPA (*n* = 7), in which 5 were able to discontinue oral steroids [194].

Newer treatment strategies targeting the type 2 inflammatory response in ABPA in CF have been evaluated for patients unable to tolerate systemic corticosteroids or unable to taper off glucocorticoids despite antifungal therapy. Monoclonal antibodies such as omalizumab, the anti-IgE monoclonal antibody, are approved for asthmatic patients ≥ 6 years old, but there is limited evidence for its use in ABPA in children with CF [170]. Retrospective observational studies in adults and children with CF and ABPA (*n* ≤ 32) have shown that omalizumab is well tolerated, but there were mixed results in terms of its effect on lung function [195,196,197,198]. A case series (*n* = 13) of children with CF and ABPA showed that omalizumab led to improvement in lung function, fewer respiratory symptoms, and reduced corticosteroid use [199]. A double-blind RCT of omalizumab (with daily injections for 6 months) in ABPA in CF patients was terminated early due to low patient recruitment and retention issues (Clinical Trials gov identifier NCT000787917) [170,200].

There have been no studies on monoclonals other than omalizumab in children with CF and ABPA. Anti-IL5 antibodies and antibodies against IL4 and IL13 have been evaluated in adult patients with ABPA, but have not yet been studied in pediatric patients [201,202,203].

**Table 3:** Studies on the management of *Aspergillus* in children and young people with cystic fibrosis.

Study Type	Number and Age of CF Patients	Management	Outcomes
*Aspergillus* colonization			
Aaron 2012 [177]: RCT (2008–2010)	*n* = 35 children and adults Mean age 25.3 years (±10.5 SD) Persistent colonization with *A. fumigatus* (≥2 positive sputum cultures in last 12 months)	Oral itraconazole versus placebo	Therapeutic blood levels of itraconazole were not achieved in 43% of patients, therefore no conclusions were drawn about its effects
*Aspergillus* colonization and Allergic bronchopulmonary aspergillosis			
Patel 2020 [173]: Single-center prospective non-randomized open-label observational study (2014–2018)	*n* = 14 children with 23 patient episodes (courses of prednisolone) Median age 13 years (range 3–17 years) 9 patient episodes for emerging or active ABPA 12 patient episodes for *Aspergillus* colonization (≥50% positive sputum samples over 6–12 months, excluding ABPA)	Oral posaconazole 3 months (13 courses oral suspension; 10 courses of tablets) 11 courses for ABPA 12 courses for *Aspergillus* colonization	Median 4–6% improvement in lung function with posaconazole in the whole cohort (*p* = 0.015) All ABPA patients and 7/12 colonized patients reported an improvement in cough Therapeutic plasma levels (>1 mg/L) were achieved in all receiving tablets, and 60% in those receiving suspension Posaconazole was well tolerated
Allergic bronchopulmonary aspergillosis			
Gothe 2020 [174]: retrospective case-control (2007–2016)	*n* = 65 children and adults with CF and ABPA and *n* = 127 children and adults with CF without ABPA (sex-matched controls with similar FEV_1_ and *Pseudomonas* status) Median age 13.2 years (IQR 9.9–20.9) (controls) and 13.5 years (IQR 10.3–23.3) (ABPA group)	Oral short course of steroids (18 days) in combination with itraconazole for 12 months	Lung function was restored to pre-ABPA levels within 3 months of treatment (*p* value < 0.0001) when compared to pretreatment No differences in long-term FEV_1_ decline in treated ABPA patients versus controls Lower itraconazole levels were associated with ABPA relapses
Thomson 2006 [187]: Case series (2000–2004)	*n* = 4 with severe ABPA (recurrent relapses or poor disease control on conventional therapy) Age range 19 months to 8 years	IV methylprednisolone 15–20 mg/Kg/day for 3 days and repeated every 4 weeks. Stopped when disease controlled or if not tolerated	Disease control achieved in 3 out of 4 children Multiple side effects of IV methylprednisolone were experienced (facial flushing, malaise, transient hyperglycemia)
Cohen-Cymberknoh 2009 [186]: Case series (2002–2008)	*n* = 9 children and adults with first episode of ABPA Mean age 17.1 years (range 7–36 years)	High-dose pulsed IV methylprednisolone 10–15 mg/Kg/day for 3 days per month with itraconazole until resolution of ABPA (6 to 10 pulses)	All patients showed clinical and laboratory improvement comparing before and after pulsed IV methylprednisolone Minor adverse effects occurred which disappeared shortly after each IV pulse therapy (excessive weight gain, transient emotional instability)
Abbotsford 2021 [191]: Single-center retrospective cohort study (2018–2020)	*n* = 16 children with ABPA Median age 12 years (range 5–16 years)	SUBA-itraconazole^®^ 10 mg/kg/d (max 400 mg/day) median duration 6 weeks	59% achieved therapeutic levels (1–2 mg/L for treatment). 81% ≥ 35% decline in IgE Well tolerated
Hilliard 2005 [171]: retrospective case note review (2002–2004)	*n* = 21 of which 13 ABPA and 9 *Aspergillus* colonization (≥1 positive culture in preceding 12 months) Median age 11.3 years, range 5–16 years	Oral voriconazole for 1 to 50 weeks (median 22 weeks)	Voriconazole associated with improved lung function in those with ABPA compared to before voriconazole treatment 7 children (33%) had adverse events (photosensitivity, nausea)
Glackin 2009 [172]: retrospective case series	*n* = 110, of which 10 had ABPA (of whom 1 died before completion of voriconazole course) Mean age of 9 surviving patients with ABPA 14.4 years (±0.2 SD)	Oral voriconazole in addition to oral steroids	Treatment with voriconazole was associated with reduced oral steroid requirement, reduced total IgE, and increased lung function One patient developed side effects of visual disturbance, photosensitivity
Hassanzad 2019 [192]: case report (2016)	12-year-old boy with CF and steroid-dependent ABPA due to *Aspergillus terreus*, unresponsive to corticosteroids and itraconazole	Oral voriconazole 3-month course	Clinical, serological, and mycological improvement after treatment course
Proesmans 2010 [194]: case series (1998–2009)	7 children and adults with CF and recurrent/difficult-to-treat ABPA with failure to taper systemic corticosteroids Age range 7 to 20 years old	Nebulized amphotericin alone or in combination with oral itraconazole or voriconazole	Nebulized amphotericin treatment was associated with withdrawal of steroid therapy without ABPA relapse in 12 months in 6 out of 7 patients (2 of these 5 patients also treated with voriconazole, though levels sub-therapeutic) and associated with improvement in lung function in 5 of the 6 patients
Nové-Josserand 2017 [198]: Case series (2008–2012)	21 adults and 11 children with CF and ABPA Median age 23 years (range 11–59 years)	Omalizumab once every 2–4 weeks	No significant improvement in lung function, BMI, or oral corticosteroid use when comparing before and after treatment
Tanou 2014 [199]: Case series (1990–2013)	Synthesis of 8 case reports of 13 children with CF and ABPA from the literature, in whom 69.2% had experienced adverse effects due to long-term steroids Mean 13.3 years (±1.5 SD), range 11 to 16.3 years old	Omalizumab once every 2–4 weeks for 1.5–32 months	Omalizumab was associated with a significant increase in lung function (ppFEV_1_) and reduction in proportion of patients receiving corticosteroids compared to pretreatment Significantly fewer patients had recurrent respiratory exacerbations after treatment
Perisson 2017 [196]: Case series	*n* = 18 children and adults with ABPA Mean age 17.1 years (±5.2 SD)	Omalizumab once every 2–4 weeks for up to 12 months	Omalizumab was associated with a stabilization of lung function decline and a significant decrease corticosteroid daily dose No serious side effects of omalizumab reported
*Aspergillus* bronchitis			
Shoseyov 2006 [117]: case series (2002–2003)	6 patients with *Aspergillus* bronchitis (AB) in CF refractory to broad-spectrum IV antibiotics. 4 children aged 10 to 15 years old and 2 adults	Oral itraconazole (1–3 months) unless otherwise specified.	10, 13, and 15-year-olds with AB: oral itraconazole led to improved lung function back to baseline 12-year-old with AB: Failed treatment with oral itraconazole. IV amphotericin B (2 weeks) then oral voriconazole (4 months) associated with lung function returning to normal

CF = cystic fibrosis; RCT = randomized control trial; SD = standard deviation; ABPA = allergic bronchopulmonary aspergillosis; IQR = interquartile range; mg/L = milligrams per liter; ppFEV_1_ = percentage predicted forced expiratory volume in 1 s; IV = intravenous; BMI = body mass index; SUBA-itraconazole^®^ = super-bioavailability itraconazole; AB = *Aspergillus* bronchitis.

## 6. Discussion

In this review, we have described the latest insights on the immunopathology of *Aspergillus* infection in pwCF and have provided a current overview of the clinical epidemiology, management, and outcome of *Aspergillus* infection in children and young people with CF. An improved understanding of antifungal immunity in pwCF has the potential to inform new management strategies with the aim to prevent lung damage and lung function decline from early age onwards.

Associating the presence of *Aspergillus* in the airways with the progression of CF lung disease has been cumbersome due to the progressive lung disease and lung function decline being intrinsically linked to the nature of CF, reverse causality, and the impact of recurrent bacterial airway infections in children with CF. In contrast to specific bacterial pathogens (e.g., *P. aeruginosa*, *Staphyloccocus aureus*), for which eradication therapies have been developed to preserve lung function early in life, this has not been considered for *A. fumigatus*. *Aspergillus* in the airways of children with CF has been interpreted for a long time as harmless colonization caused by the inhalation of airborne conidia being trapped in the sticky mucus. Still, the clinical significance of *Aspergillus* colonization, either intermittent or persistent, is not well understood. Age of acquisition seems to play a role, with being colonized at a younger age (<6 years of age) having a negative impact on CF lung disease and pulmonary exacerbations [9,20]. Our recent longitudinal study, which included 1675 children and young people with CF aged 8 to 18 years of age, found *Aspergillus* colonization did not show any long-term effect on lung function [115].

In the last few years, the clinical landscape of CF has changed enormously, with 73% of UK CF patients on CFTR modulator therapy in 2023 (64% on elexacaftor/tezacaftor/ivacaftor (ETI)) [6]. No real-world studies have been published on the impact of the rollout of combination CFTR modulator therapy on the frequency of *Aspergillus* and ABPA, though in clinical trials ivacaftor led to a reduction in *Aspergillus* colonization [110,204]. Preliminary data from a single-center retrospective observational study show that ETI combination CFTR modulator therapy led to reduced *Aspergillus*-positive sputum cultures [205]. The underlying mechanisms by which CFTR modulators reduce *Aspergillus* infection and disease is an area of interest, with the reduction in *Aspergillus*-induced ROS from CF phagocytes thought to play a key role [69]. The gradual reduction in prevalence of positive *Aspergillus* respiratory samples and ABPA since 2019 reported in European and UK CF registry databases [1,6] correlates with the widespread availability of combination CFTR modulator therapy.

As the significance of *Aspergillus* colonization is not yet well understood, management strategies vary greatly [168,176]. Clinical guidelines vary significantly on whether to treat persistent *Aspergillus* colonization with azole therapy, including the type of azole, length of azole therapy, and monitoring for clearance and side effects [175,206]. It is unlikely that a further randomized controlled trial on the use of azoles for *Aspergillus* colonization in children with CF will be feasible given that previous randomized controlled trials on this were terminated early due to challenging patient recruitment [177,178]. Now that detailed real-world electronic patient data are increasingly available, there is the opportunity to evaluate different azole treatment protocols in the context of CFTR modulator treatment. The RECOVER and CF Foundation studies from the UK and USA are prime examples of this [207,208]. One important area that requires further study is the drug–drug interaction between CFTR modulators and azoles, as azoles inhibit cytochrome P450 (CYP) 3A4-mediated biotransformation, thus causing increased exposure to CFTR modulator therapy and requiring a dose reduction of CFTR modulator therapy [178,188,189]. Clinical trials to evaluate the optimal treatment protocols for children on CFTR modulators and azole therapy, as well as minimizing steroid exposure, are required. Novel approaches in the use of immunomodulatory treatment to target the host–pathogen interaction have shown some promising early results in ex vivo studies [58,69,104,209].

Future targeted therapies are likely to arise from a detailed understanding of the underlying immunopathology of *Aspergillus* infection in the context of CFTR modulator therapy, focusing on suppressing or augmenting the immunological response to *Aspergillus*. Key factors that need to be considered when planning trials of treatments for *Aspergillus* in children with CF in the CFTR modulator era include management of drug–drug interactions and optimal clinical endpoints.

Here, we have provided a summary of current understanding of the immunopathology, clinical features, and management of *Aspergillus* in children with CF, and presented the limitations of our current knowledge. We have highlighted the need to harness real-world data to understand the impact of CFTR modulator treatment on allergic and non-allergic *Aspergillus* disease in CF, and to further evaluate the optimum management strategies of *Aspergillus* to ultimately lead to improved patient outcomes.
